# Exploring neurologists’ perspectives on the return of next generation sequencing results to their patients: a needed step in the development of guidelines

**DOI:** 10.1186/s12910-018-0320-3

**Published:** 2018-09-29

**Authors:** Thierry Hurlimann, Iris Jaitovich Groisman, Béatrice Godard

**Affiliations:** 1 Institut de recherche en santé publique, Université de Montréal, PO Box 6128, Station Centre-ville, Montreal, Quebec, H3C 3J7 Canada; 2 Department of Social and Preventive Medicine, Université de Montréal, PO Box 6128, Station Centre-ville, Montreal, Quebec, H3C 3J7 Canada; 3 Quebec Population Health Research Network, PO Box 6128, Station Centre-ville, Montreal, Quebec, H3C 3J7 Canada

**Keywords:** Whole Genome Sequencing, Neurological disorders, Incidental findings, Recontacting patients, Clinical utility, Ethics

## Abstract

**Background:**

The use of Next Generation Sequencing such as Whole Genome Sequencing (WGS) is a promising step towards a better understanding and treatment of neurological diseases. WGS can result into unexpected information (incidental findings, IFs), and information with uncertain clinical significance. In the context of a Genome Canada project on ‘Personalized Medicine in the Treatment of Epilepsy’, we intended to address these challenges surveying neurologists’ opinions about the type of results that should be returned, and their professional responsibility toward recontacting patients regarding new discovered mutations.

**Methods:**

Potential participants were contacted through professional organizations or direct invitations.

**Results:**

A total of 204 neurologists were recruited. Fifty nine percent indicated that to be conveyed, WGS results should have a demonstrated clinical utility for diagnosis, prognosis or treatment. Yet, 41% deemed appropriate to return results without clinical utility, when they could impact patients’ reproductive decisions, or on patients’ request. Current use of targeted genetic testing and age of patients influenced respondents’ answers. Respondents stated that analysis of genomics data resulting from WGS should be limited to the genes likely to be relevant for the patient’s specific medical condition (69%), so as to limit IFs. Respondents felt responsible to recontact patients and inform them about newly discovered mutations related to the medical condition that triggered the test (75%) for as long as they are following up on the patient (55%). Finally, 53.5% of the respondents felt responsible to recontact and inform patients of clinically significant, newly discovered IFs.

**Conclusion:**

Our results show the importance of formulating professional guidelines sensitive to the various – and sometimes opposite – viewpoints that may prevail within a same community of practice, as well as flexible so as to be attuned to the characteristics of the neurological conditions that triggered a WGS.

## Background

Next Generation Sequencing technologies (NGS) – mainly whole-genome sequencing (WGS) and whole-exome sequencing (WES) – are being integrated into clinical practice because they improve the characterization and diagnosis of a wide variety of medical conditions [1]. The use of WGS and WES increases the probability of obtaining information unrelated to the circumstances that triggered the need for a genetic diagnosis as it delivers more complete and broader information than traditional sequencing [1, 2]. The acquisition of such unrelated information – commonly known as incidental findings (IFs) – in clinical settings has been the object of intense debate at various professional levels worldwide, with different professional bodies issuing recommendations [3–7]. Topics of discussion range from utility and validity of the obtained information, treatment availability for newly found conditions, age of onset of a disorder and professional liability with respect to the communication – or not – of unpredicted results, among others [8, 9]. Recent empirical work highlights the relevance of stakeholders’ beliefs and expectations on the matter of conveying IFs [5, 10].

Neurological conditions have highly heterogeneous etiologies and for many a genetic basis has been described [11–15]. NGS technologies such as WGS and WES offer a powerful tool in research to potentially improve diagnosis, prognosis, as well as clinical personalized treatment of neurological conditions [16–18]. At the same time, targeted genetic testing for neurological disorders has been used for specific conditions and in different contexts. For instance, in the case of Huntington disease [HD], one affected gene presents a short unstable sequence which varies in number in different cases of HD. This variation in number represents valuable information for family members of the affected individual, underlines the important involvement of genetic counseling for the entire family, and then justifies the need of guidelines for genetic testing for HD to help in testing and informing results [19]. In other cases, targeted genetic testing for neurological conditions has evolved from testing a single gene to a panel of genes to the use of WES to diagnose - for instance -, amyotrophic lateral sclerosis [20]. As for Alzheimer Disease (AD), the knowledge has progressed from identifying three genes as a cause of autosomal dominant AD (a minority of AD cases) to more than twenty for the genetically complex form of AD [21]. Thus, the knowledge of new genetic information – including IFs – complicates the interpretation of results, increases the need for genetic counseling before and after the test for a large number of individuals, and triggers the need to update disease management guidelines [20, 21]. While some professional guidelines such as the ones issued by the Canadian College of Medical Geneticists on the management of monogenic diseases in Canada “do not endorse the intentional clinical analysis of disease-associated genes other than those linked to the primary indication” [7], p., 431, the increasing identification of new gene variants through WES and WGS seems unavoidable even for already known disease-associated genes.

The intertwined risks and benefits that genetic information – related or unrelated to the medical condition triggering the test – may bring to patients with various neurological conditions merit a specific discussion. On the one hand, genetic results can impact patients’ lives for instance by improving health preventive measures. But at the same time, it can make it difficult to obtain health insurance coverage for family members and/or increase social and psychological risks, such as anxiety, stigma or discrimination in a population that may already be vulnerable due to their neurological medical condition. On the other hand, conveying this type of information is a new undertaking for neurologists, which would probably have to be carried out without clear accompanying professional guidelines.

The work we present herein is part of a Genome Canada project on ‘Personalized Medicine in the Treatment of Epilepsy’, which aimed to determine whether WGS could be used as a valuable “diagnostic tool”, in particular for pharmaco-resistant epilepsies and to find out the genetic cause of the condition as well as the response - or lack thereof - to medication [22]. In this context, we deemed it important to explore neurologists’ opinions about the return of WGS results in their medical practice – including unexpected ones –, their commitment to stay in contact with patients as new information may develop, and their views on patients’ right to know and being informed of results, even those with an uncertain clinical significance. An earlier publication presented information derived from the same study addressing neurologists’ positions and perceived risks and benefits on the use of WGS, as well as the infrastructure (i.e., training, guidelines) they may need to incorporate WGS in their practice [23]. The present work provides original information on returning of genetic results and neurologists’ responsibility to recontact patients.

Our findings can contribute to clearer guidelines for neurologists on returning genetic results to their patients thus facilitating the implementation of WGS and other NGS technologies in their clinical practice.

## Methods

### Recruitment and data collection

Neurologists worldwide were contacted through professional associations at international, regional, and national levels. A total of 190 neurology societies and associations in 215 countries/geographical areas were identified and contacted. The list of countries/geographical areas was based on the World Health Organization (WHO) regions (http://www.who.int/countries/fr/). The latter were asked to distribute an invitation to participate in our study. A letter was simultaneously sent to 830 neurologists whose coordinates were publicly available on association websites. Invitations were also sent by email to 581 corresponding authors in clinical neurological research worldwide, identified through PubMed for the years 2012–2014. Neurologists specialized in the treatment of epilepsy registered with the International League Against Epilepsy’s website were directly contacted as well, adding 260 neurologists to the worldwide contact list. We likewise asked participants to forward the invitation to colleagues. The challenges faced in the recruitment of neurologists worldwide, as well as the complete procedures used to reach them are described in detail in a previous publication [24]. Online questionnaires and data collection were conducted by the IT department at Université de Montréal’s Public Health Research Institute (IRSPUM). Data collection was anonymous to the researchers. The study was approved by the Research Ethics Board of the research ethics committee at the Université de Montréal and of Centre hospitalier de l’Université de Montréal (CHUM).

The resulting sample consisted of 204 neurologists based in Europe, North, Central and South America, the Caribbean, South-East Asia, the Western Pacific, and in Eastern Mediterranean Regions. Among the 204 neurologists who completed the survey, almost half treated mostly adult patients (48%) while the rest treated mainly children (37.3%) or both groups of patients (14.7%). As for medical conditions, epilepsy (73%) and headaches 112 (57.8%) were those predominantly treated [23].

### Survey – Instrument and data analysis

The survey questionnaire contained questions aimed at exploring neurologists’ views and perspectives on: (1) clinical practices with genetic/genomic testing (including WGS); (2) circumstances and/or conditions in which WGS should be offered to patients; (3) the potential benefits of the use of WGS; (4) concerns about the use of WGS, (5) the return of results; and (6) needs for training or resources in genomics/genetics [23, 24]. This manuscript only focuses on neurologists’ views on the return of results and on their responsibility to recontact their patients as new mutations are discovered over time.

Data analysis has been previously described [23]. Briefly, coding and analysis were conducted using the Statistical Package for Social Sciences (SPSS) software, v. 23.0. Descriptive statistics were used to portray sample characteristics. The interpretative analysis, based on χ2 and factor analyses, allowed us to better characterize the respondents and to reach a deeper understanding of what motivated and guided their responses. Two profiles emerged: (a) neurologists who would offer WGS to their patients and (b) those who would not offer WGS to their patients, or did not know about the uses of WGS. A one-way ANOVA was conducted to compare the two profiles. We considered a *P*-value of 0.05 or less as statistically significant.

## Results

### Returning genetic results

Table 1 presents the comprehensive outcomes of the survey on conveying genetics results to patients. We asked neurologists to choose among two statements that most closely described their opinion about the return of WGS results, including IFs. Over half of respondents to these questions (*n* = 94/159, 59.1%) indicated that to be returned to a patient, WGS results should always have a demonstrated clinical utility for diagnosis, prognosis or treatment. Less than half of respondents (*n* = 65/159, 40.9%) indicated that in some circumstances it may be appropriate to return WGS results – directly related or not to the matter that prompted the test – even without demonstrable clinical utility. The latter largely agreed that results that could impact patients’ or proxies’ reproductive decisions (*N* = 50/63, 79.4%) and/or an explicit request from parents or proxies to be informed of WGS results (*N* = 49/63, 77.8%) were strong enough reasons to return WGS results, even if their clinical significance was uncertain (Table 1).

**Table 1:** Neurologists’ perceptions on returning WGS results

Questions and number of respondents	Percentage
**Gender** (*n* = 155)	
Female	51.6
Male	48.4
No answer	24.0
**Characteristics of patients** (*n* = 204)	
Mainly Adults (*n* = 98)	48.0
Mainly Children (*n* = 76)	37.3
Both (*n* = 30)	14.7
**Which of the following statements most closely describes your opinion about the return of WGS results to the patient, including incidental findings?**	
a. To be returned to a patient, WGS results should always have a demonstrated clinical utility for diagnosis, prognosis or treatment (*n* = 94/159)	59.1
b. In some circumstances it may be appropriate to return WGS results that do not have a demonstrated clinical utility for diagnosis, prognosis or treatment (*n* = 65/159)	40.9
i. reproductive decisions (*n* = 50/63)	79.4
ii. parents’/proxies’ requests (*n* = 49/63)	77.8
**To be returned to a patient, an incidental finding should always…**(statements were not mutually exclusive)	
a. Be related to a disease for which effective preventive interventions or treatments were available (*n* = 111/158)	70.3
b. Indicate a high risk of developing a specific disease (*n* = 104/158)	76.8
c. Be about a disease whose onset may be expected in a near future (as opposed, for instance, to a result indicating a risk for a child of developing a disease late in adult life only) (*n* = 77/158)	50.0
**You agree that in WGS, analysis of the genomics should be limited to the genes likely to be relevant for a patient’s specific medical condition, so as to limit IFs** (*n* = 108/157)	68.8

As to incidental findings (IFs) exclusively, we asked neurologists which characteristics an unexpected genetic result obtained through WGS should have to be returned to a patient. Participants could select among various statements that were not mutually exclusive (Table 1). A majority of respondents (*N* = 104/158, 76.8%) agreed that an IF had to indicate a high risk of developing a specific disease in order to be conveyed, and that (*N* = 111/158, 70.3%) an IF should be related to a disease for which effective preventive interventions or treatments were available. Half of respondents agreed (*N* = 77/158, 50%) that to be returned to a patient, an unexpected result should always be about a disease whose onset may be expected in a near future, as opposed, for instance, to a result indicating a risk for a child of developing a disease late in adult life only.

In certain cases, current use of genetic testing by respondents, and age of patients they most frequently followed, had an influence on their answers about the circumstances in which IFs should be returned to patients. It appeared that respondents who never used targeted genetic testing in their practice had a greater tendency to recognize that they did actually not know if an IF had to have a clinical utility to be conveyed to a patient (*p* < .05). Similarly, those using targeted genetic testing tended to agree that IFs had to show a high probability of onset for a specific disease, as opposed to a low probability (*p* < .05). Respondents who did not use targeted genetic testing were more prone to acknowledge they did not know whether such a condition of return of results had to be fulfilled or not (*p* < .05). When asked about their opinion on returning IFs only for a disease whose onset could be expected in the near future, neurologists that had already used targeted sequencing mostly disagreed with this position in comparison to those who did not use this type of genetic test (*p* < .05). In regard to the impact of the age of patients on participants’ answers, neurologists treating mostly children, when compared to those treating mainly adults, tended to disagree in greater proportion that parents or other proxies should remain free to choose not to know about IFs, including IFs about preventable or treatable diseases (*p* < .05). Male respondents tended to agree with this statement (*p* < .001), while female neurologists tended to disagree (*p* < .05).

Participants were also asked if the analysis of the genomics data resulting from a WGS should be limited to the genes likely to be relevant for a patient’s specific medical condition and associated comorbidities, so as to limit IFs. From a total of 157 participants addressing this question, a majority agreed with this view (*N* = 108/157, 68.8%) (Table 1).

### Responsibility for recontacting patients

We asked neurologists if those using WGS have a responsibility to recontact and inform their patients of clinically significant newly discovered mutations that are linked to the neurological condition that triggered the test. Table 2 shows that three-quarters of respondents feel responsible to do so. A very low fraction did not perceive it as a responsibility, while almost one in five respondents did not know what they would or should do. Those who agreed on the need to recontact were asked for how long they should be responsible to recontact a patient. More than half of respondents would do so for as long as they are following the patient. Just over one in four respondents would recontact for an indefinite amount of time, and a minority would do so for a limited period of time (Table 2). Still 13% did not know, nor were not sure what they would do. Finally, more than half of the respondents considered that they also had a responsibility to recontact and inform patients of clinically significant, newly discovered mutations that were not linked to their neurological condition (i.e., IFs), while 20% did not know whether they would have such a responsibility (Table 2).

**Table 2:** Neurologists’ perceptions about recontacting patients to inform about newly acquired genetic information

**Should neurologists who use WGS have a responsibility to recontact and inform their patients of clinically significant newly discovered mutations that are linked to the neurological condition**	
a. Yes (*n* = 118/158)	74.7
b. No (*n* = 10/158)	6.3
c. I don’t know (*n* = 30/158)	19
If yes for how long?	
a. as long as they are following the patient (*n* = 64/116)	55.2
b. for an indefinite amount of time (*n* = 32/116)	27.6
c. for a limited period of time (*n* = 5/116)	4.3
d. I don’t know (*n* = 15/116)	12.9
**Do you think that neurologists also have a responsibility to recontact and inform patients of clinically significant, newly discovered mutations that were not linked to their neurological condition**	
a. Yes (*n* = 61/114)	53.5
b. No (*n* = 30/114)	26.3
c. I don’t know (*n* = 23/114)	20.2

### Two profiles of respondents and their position on conveying IFs and recontacting patients

As presented in an earlier publication, based on the circumstances in which respondents considered offering WGS to their patients, a factor analysis brought out two neurologist profiles [23]. ‘Profile A’ (58.5%) included those who would offer WGS to all their patients and those who would do so in limited circumstances. ‘Profile B’ (31.8%) would not offer WGS or were not sure in which circumstances WGS should be offered. Profile A was characterized – in comparison to Profile B – by neurologists treating mainly children, mostly for epilepsy, and having 20 years of medical practice or less. As shown on Table 3, both Profile A and B respondents agreed that to be returned to a patient, an IF should always be about a disease for which effective preventive interventions or treatment were available. Compared to respondents in Profile B, those of Profile A tended to agree to a greater extent that results related to an unexpected condition should indicate a high probability of occurrence if they were to be communicated (*p* < .001). Similarly, Profile A respondents tended to consider that, to be returned to a patient, an IF should be about a disease whose onset may be expected in the near future, as opposed, for instance, to a result indicating a risk for a child of developing a disease late in adult life only (*p* < .001). Compared to those of Profile A, Profile B respondents were more likely to agree that parents, or other proxies, should remain free to choose not to know about IFs, including those that pertained to treatable and/or preventable conditions (*p* < .001).

**Table 3:** Two profiles of respondents

***Profiling respondents…*** ^a^	
**Profile A** (*n* = 120; 58.5%)	**Profile B** (*n* = 65; 31.8%)
- Would offer WGS to all their patients or do so in limited circumstances	- Would not offer WGS to their patients, or did not know about the uses of WGS
- Treating mainly children	- Treating mainly adults
***Returning WGS results to patients by Profile***	
- An unexpected result should always be about a disease for which effective preventive interventions or treatment are available	
*No difference between profiles*	
- An unexpected result should always indicate a high probability to develop a specific disease (as opposed to a result indicating a low probability to develop a disease	
*Profile A tended to agree (p < .001)*	
- An unexpected result should always be about a disease whose onset may be expected in a near future	
*Profile A tended to agree (p < .001)*	
- Parents or other proxies should remain free to choose not to know about unexpected finding in the patient’s W GS, including findings about preventable or treatable disease	
*Profile B tended to agree (p < .001)*	
- Conveying genetic results with uncertain clinical significance if requested by parents or proxies	
*Profile A respondents were more inclined (p < .001)*	
- Analysis of genomics data should be limited to the genes likely to be relevant to the patient’s specific medical condition, so as to limit unexpected findings	
*Profile A tended to agree (p < .001)*	
***Recontacting patients by Profile***	
- Neurologists have the responsibility to recontact and inform their patients of clinically significant, newly discovered mutations that were linked to their neurological condition	
*Profile A tended to agree (p < .001)*	
- Neurologists have the responsibility to recontact and inform their patients of clinically significant, newly discovered and for IFs	
*Profile A tended to agree (p < .05)*	
- The responsibility to return results should be for as long as neurologists are treating the patient	
*Profile A tended to agree (p < .05)*	

^a^Based on χ2 and factor analyses. A one-way ANOVA was conducted to compare the two profiles. We considered a P-value of 0.05 or less as statistically significant [23]

On the matter of conveying genetic results with uncertain clinical significance if requested by parents or proxies, Profile A respondents were more inclined to do so, compared to Profile B ones (*p* < .001). Lastly, compared to respondents in Profile B, those in Profile A were more prone to accept that analysis of genomics data should be limited to the genes likely to be relevant to the patient’s specific medical condition, so as to limit unexpected findings (*p* < .001).

On the matter of recontacting patients, most respondents in Profile A, in comparison to those in Profile B tended to believe that neurologists had a responsibility to recontact and inform their patients of clinically significant, newly discovered mutations that were linked to their neurological condition (*p* < .001), and for IFs (*p* < .05). They also believed that the responsibility to return results should be for as long as they are treating the patient.

## Discussion

### Returning results

The majority of neurologists participating in our survey agreed with the commonly accepted position that to be returned to a patient, WGS results should always have a demonstrated clinical utility for diagnosis, prognosis or treatment [25]. Still, a non-negligible percentage of respondents considered that in some circumstances, such as an explicit request from patients or their proxies, or in cases where the information could influence patients’ or proxies’ reproductive decisions, it may be appropriate to convey this information even when it did not have a demonstrated clinical utility. One of our respondent explicitly mentioned that “[d]octors should avoid paternalism, the patient is entitled to know about all the findings – but patients should be thoroughly informed of possible consequences of testing, and be well prepared psychologically before any test is carried out”. Another participant stressed that “[i]f you have the information, so should the patient”. At the same time, several neurologists agreed that parents and proxies should remain free to choose not to know about IFs, including findings about preventable or treatable diseases. A more paternalistic position was expressed by those treating mainly children. A similar stance was shown by female respondents of the whole sample, which suggests that gender may also influence opinions on patients’ or proxies’ right to know or not to know.

In a research context, Wynn and colleagues showed that genetic researchers who were not involved into the clinical practice were in general more inclined to offer return of IFs than clinicians [26]. Our results also indicated that physicians’ knowledge and background influence their positions on conveying genetic information to their patients [23]. Yet, it is a challenging endeavor to assess the actual impact of genetic knowledge and clinical experience on various health professionals’ views, as it may depend on other characteristics such as socio-economic context, personal involvement in research activities or training, and even gender as showed by our results.

There was also no agreement among respondents about conveying IFs on a given condition in relation to its age of onset. Late onset conditions do not seem to be widely discussed in the current guidelines on the return of IFs [27]. Yet, neurological conditions characterized by an early onset (i.e., affecting mostly children) may have a different impact on patients’ and families’ lives in terms of stigma and social development, as well as reproductive decisions, compared to those conditions that appear later in the life of an individual. Earlier results from our survey showed that respondents expressed concerns about the impact of WGS on their patients and immediate families regarding communication of results, understanding of information, and the risks of discrimination and stigmatization [23].

In their survey, Yu et al. showed that genetics professionals were split “as to whether only actionable results (from WGS and WES) should be offered for return” [28]. In addition, participants in Yu and al.’s study agreed on the significance to be given to patients’ and/or their families’ preferences on the type of IFs that should be returned. Neurologists’ perspectives on this matter are similar in our survey.

The information obtained from our study and ones conducted elsewhere might indicate some common ground on facilitating a previously proposed partnership between physician and patient to preserve patients’ autonomy to decide on the type of information to be received [29]. However, efforts should initially be made to establish common definitions for clinical utility/actionability, as discussed by Moret et al. [9]. In the context of genetic research in psychiatry, Lázaro-Muñoz and colleagues recommended that genomic results (including IFs) that could help corroborate or reject a psychiatric diagnosis be offered to participants, even if not medically actionable, by introducing the terms “clinically valuable and likely clinically valuable findings” [30]. Such a recommendation illustrates the difficulties to define a common concept of “actionability”, be it in different settings (i.e., clinics vs. research), or for a specific group of medical conditions (neurology vs. psychiatry). It is crucial to acknowledge that patients’ and/or proxies’ views on what constitutes an “actionable” or “useful” result may significantly differ from a clinical perspective on validity or utility, an issue that has been debated in terms of “personal utility” [31–33].

Our respondents have thus shown divergent opinions about patients opting for not receiving genetic information, and on returning IFs with or without clinical validity. These results echo the heterogeneity of current positions/regulations of various medical associations on the management of incidental findings [27]. For instance, the American College of Medical Genetics and Genomics allows an opt-out possibility by parents on behalf of their children, in contrast to the American Society of Human Genetics, the Canadian College of Medical Geneticists and the Public and Professional Policy Committee of the European Society of Human Genetics, all of which have more protective attitudes towards minors [27]. The recommendations on returning IFs are diverse and sometimes even contradictory. This can lead to confusion and even concerns among health professionals. As shown in an earlier publication, neurologists who are currently using genetic tests in their practice are concerned about their legal responsibility when providing (or not) genetic results and IFs [23]. Such concerns are likely due to a lack of guidance and/or clarity on the course of action they should propose. Recommendations issued by the American College of Medical Genetics and Genomics on the return of IFs triggered an analysis of the new legal responsibilities to which health professionals are exposed, involving compensation for any damages or injuries suffered when conveying – or not – such information [34]. Knoppers et al., in a review of the current legislative landscape for WGS-based genetic testing in the US, Canada and Europe, proposed that policies should be at least specific to the characteristics of the “clinical domains” that the findings address [27]. Based on the latter and on the results of our study, policies regulating the return of genetic results and IFs in neurology would have to take into consideration – in order to better serve and protect all stakeholders – the opinions of members of professional associations, and the importance the input of patients and proxies may have for neurologists. There are calls for further empirical research on that matter among physicians of various backgrounds and specialties, as it could be helpful in informing future regulations that would better preserve the autonomy of patients’ and proxies’ decisions.

### Recontacting patients

A recent systematic review on recontacting patients about new genetic information shows an increased discussion in light of NGS technologies [35]. There are various issues surrounding the recontacting of patients in order to provide them with new genetic information, including determining the scenarios in which recontacting should occur, ethical implications of contacting patients out of schedule, patients’ well-being and physicians’ liability [35]. The majority of neurologists in our survey felt compelled to recontact their patients with updated genetic information linked to the medical condition that triggered the test. However, we were surprised by the significant percentage of respondents who agreed to be held responsible to return information about newly discovered mutations that are not related to their patients’ neurological condition. Indeed, management of such unexpected findings in time involve important organizational aspects and should include the availability of multidisciplinary teams and a health system capable of managing additional diagnostic procedures and treatments for unforeseen medical conditions [36]. Yet, a large percentage of our respondents also supported the idea that WGS should be limited to the genes likely to be relevant for a patient’s specific medical condition and associated comorbidities, so as to limit IFs. This stance does clearly reduce the instances when neurologists would agree to face such a duty to recontact patients to convey information about IFs. As presented in a former publication, respondents in our survey expressed a need for explicit guidelines from professional organizations in regulating the use of NGS technologies in their medical practice [23]. However, the results show that they do not share the same views on returning results in terms of clinical actionability and in terms of recontacting to inform about IFs. While guidance is certainly needed to ensure patients’ well-being, as well as to clarify physicians’ legal responsibilities, those involved in the development of guidelines face a challenging task and should not overlook factors that may influence health professionals’ perceptions, such as gender, age of patients, current use of genetic testing, as shown in our results.

## Limitations

As explained in a previous publication that reported other original data from the same population sample [23], our study sought to include the opinions of neurologists worldwide and although invitations were issued in English, French, and Spanish, we acknowledge that presenting our survey in English exclusively may have prevented broader participation. In addition to the language barrier, expertise, access to genetic testing, as well as cultural background may have ultimately influenced the decision to participate, or not, in our survey. Our results represent those neurologists who actually responded and thus are interested in the subject of this survey and wanted their voices to be heard. A higher rate of participation in general, and a more prominent representation of neurologists with specialties other than epilepsy, might have led to different results. In addition, given the worldwide scope of our study, we would have wanted to take into account factors such as accessibility to WGS, as well as ethical, cultural, and economical characteristics in different societies for analysis, which we could not pursue due to the limited number of participants per country or even per geographical region.

Lastly, actual participation rates could not be calculated. We could not get information about the number of members of every participating association and had no control over the means used by these organizations to disseminate our invitation (mass email to members, invitation published in a newsletter or on a website). Participation rates could clearly be impacted by these means of dissemination, as well as by snowball sampling procedures and the fact that some countries were more (or less) represented in the 1671 invitations we directly sent to neurologists in an attempt to improve the overall participation rate. Nevertheless, the present study contributes to the understanding of neurologists’ opinions surrounding new sequencing technologies and the return of results to their patients. It is worth noting that the recruitment of health professionals has always been challenging, and response rates vary widely in this population, depending on numerous factors. This challenge was further addressed and discussed in a previous publication [24]. Although Web-based surveys are useful tools for timely and worldwide responses, additional measures need to be employed in order to increase the response rate and encourage participation. In a recent study, it has been shown that neither emphasizing a monetary incentive in the subject line of the invitation email nor varying the sending day of the invitation email increased the response rate in a Web-based trial with primary care physicians [37]. The authors recommend further studies to find an effective invitational strategy using multimodal media beside email to improve response rates of physicians in Web-based studies. Based on our approach with professional organizations, we could also recommend among additional measures to encourage participation awareness campaigns, especially with professional organizations.

## Conclusion

While further studies are being conducted and professional guidelines have been evolving, most neurologists would probably prefer – as indicated by participants of this survey – to be confronted with the least amount of IFs as possible. Yet, it is imperative to address the formulation of new, practical WGS guidelines according to the characteristics of medical areas and subspecialties, as well as to local healthcare policies and patients’ health coverage. Our results show that such guidelines should also be sensitive to the various – and sometimes opposite – viewpoints that may prevail within a same community of practice, such as neurology. In such circumstances, guidelines should be flexible so as to be attuned to the reality of medical practice, but also to the characteristics of the medical conditions that triggered a WGS, and health professionals’ perspectives. By the same token, guidelines should consider the growing number of studies that assessed patients’ preferences in this field. Taken altogether, this is clearly a demanding task.

## Abbreviations

IFs: Incidental findings
NGS: Next Generation Sequencing technologies
WES: Whole exome sequencing
WGS: Whole Genome Sequencing
